# Professor Owen’s Opinions upon the Unity and Antiquity of the Human Race

**Published:** 1854-07

**Authors:** 


*Professor Owen's Opinions upon the Unity and Antiquity of the Human Race.*

In conclusion, it only remains to offer a few words respecting the antiquity of the *quadrumana* and of man upon the surface of the earth. At the time of the demise of Baron Cuvier, in 1832, no evidence had been obtained of fossil *quadrumana,* and the baron supposed that both these and the *bimana* were of recent introduction. Soon after the loss of that great reconstructor of extinct species, evidence with regard to the fossil *quadrumana* was obtained from different quarters. In the oldest (*eocene*) tertiary deposits in Suffolk, specimens of jaws and teeth were found that unerringly indicated the former existence of a species of monkey of the genus *Macacus* (*Macacus eocenus.*) About the same time the tertiary deposits from the Himalayan mountains gave further evidence of the *quadrumana,* jaws, astragali, and some other parts of the skeleton, having been found completely petrified, and referable to the genus called *Semnopithecus,* which is now restricted to the Asiatic Continent. Dr. Lund discovered in Brazil remains of an extinct platyrhine monkey, surpassing any known *cebus* or *mycetes* in size. Lastly, in the middle tertiary series in the south of France, was discovered a fragment of the lower jaw, proving that at that period some species of the long-armed ape (*Hylobates*) must have existed. But no human remains have been found in the regularly deposited layers of any of the divisions (not even the pliocene) of the tertiary series. Human bones have been found in doubtful positions, geologically considered, such as deserted mines and caves, but never in tranquil undisturbed deposits, participating in the mineral characters of the undoubted fossils of those deposits. The petrified negro skeletons in the calcareous concretes of Guadaloupe are of comparatively recent origin.

Thus, therefore, in reference both to the unity of man's species, and the fact of his being the latest, as he is the highest, of all animal forms upon our planet, the interpretations of God's works coincide with what has been revealed to us as to our own nature and origin in his Word. Of the nature of the creative acts by which the successive races of animals were called into being we are ignorant. But this we know, that, as the evidence of unity of plan testifies to the oneness of the Creator, so the modifications of the plan for different modes of existence illustrate the beneficence of the Designer. Those structures, moreover, which are at present incomprehensible, as adaptations to a special end, are made comprehensible on a higher principle, and a final purpose is gained in relation to human intelligence; for in the instances where the analogy of humanly invented machines fails to explain the structure of a divinely created organ, such organ does not exist in vain, if its truer comprehension in relation to the Divine idea lead rational beings to a better conception of their own origin and Creator.—*London Med. Times.*

